# Involvement of RUVBL1 in WNT/*β*-Catenin Signaling in Oral Squamous Cell Carcinoma

**DOI:** 10.1155/2022/3398492

**Published:** 2022-04-22

**Authors:** Yongfa Zeng, Ying Kong, Lan Liao, Hongshui Zhu

**Affiliations:** ^1^The Affiliated Stomatological Hospital of Nanchang University, The Key Laboratory of Oral Biomedicine, Jiangxi Province, Jiangxi Province Clinical Research Center for Oral Diseases, Nanchang City, 330006, Jiangxi Province, China; ^2^The First Affiliated Hospital of Nanchang University, Nanchang City, 330006, Jiangxi Province, China

## Abstract

Oral squamous cell carcinoma (OSCC) is the most common malignant tumor of head and neck squamous cell carcinoma (HNSCC), but the causes and molecular mechanisms remain unclear. The wingless-integrated/*β*-catenin (WNT/*β*-catenin) signaling pathway plays a vital role in cancer cell proliferation, differentiation, and metastasis, including OSCC. To screen potential *β*-catenin-associated genes involved in OSCC, the intersection of these genes in the STRING and IMEx databases was assessed using differential expression genes (DEG) from public microarrays, and 22 were further selected to construct a *β*-catenin-protein interaction network. The top 14 hub genes (node degree > 10) within the network were selected. Pearson's correlation analysis showed that *β*-catenin expression correlated positively with the expression of 11 genes, including *AR*, *BIRC5*, *CDK6*, *DKK1*, *GSK3B*, *MET*, *MITF*, *PARD3*, *RUVBL1*, *SLC9A3R1*, and *SMAD7*. A heat map of overall hub gene survival was created, and elevated expression of DKK1 and RUVBL1 was associated with poor survival using the Mantel-Cox test. To identify the function of RUVBL1, colony formation assay, transwell assay, and western blotting revealed that knock-down of RUVBL1 by siRNA decreased H157 and Cal-27 cell proliferation and metastasis by inhibiting *β*-catenin signaling. These findings suggest that RUVBL1 may serve as a diagnostic and prognostic biomarker for OSCC, as well as a therapeutic target, and may help to uncover additional molecular mechanisms of *β*-catenin-driven OSCC tumorigenesis.

## 1. Introduction

Oral squamous cell carcinoma (OSCC) is the most frequent malignant tumor of head and neck squamous cell carcinoma (HNSCC) [1]. Incidence and mortality have increased in recent years worldwide [2]. At the same time, many of the causes and mechanisms underlying OSCC progression remain unclear. The wingless-integrated/beta-catenin (WNT/*β*-catenin) signaling pathway is composed of ligands, receptors, and coreceptors that are associated with signal transduction and induce a variety of intracellular responses. Aberrant activation of WNT/*β*-catenin signaling is found in many types of tumors and plays an intrinsic role in cancer cell proliferation, differentiation, metastasis, and other functions [3].

WNT/*β*-catenin signaling is considered the most important signaling pathway in OSCC [3]. Several hub genes involved in WNT/*β*-catenin signaling pathway have been identified as therapeutic targets and diagnostic biomarkers of OSCC [3], including DAB2 [4], Msi2 [5], E-cadherin, and *β*-catenin [6]. Alteration of membrane and nuclear expression of *β*-catenin are closely correlated with differentiation, invasion, and poor prognosis [7, 8]. In addition, the shift from membrane to cytoplasmic expression of E-cadherin (73.3%) and *β*-catenin (78.3%) in OSCC correlates with histologic grade [9]. Other studies have shown that nuclear *β*-catenin staining is a potential prognostic marker for ovarian cancer in univariate analysis, while cytoplasmic and membranous *β*-catenin staining is not [10]. The epithelial-mesenchymal transition (EMT) is a vital process in cancer development and progression. Cancer cells experiencing EMT can migrate from the original tissue and invade stromal tissues and more distant regions [11]. OSCC tissue has an active EMT phenotype as compared with normal oral mucosa. This phenotype includes decreased E-cadherin and increased N-cadherin and *β*-catenin expression in the nucleus [9, 12]. Thus, it is important to conduct a thorough inquiry into the relationship between *β*-catenin and the related gene expression and clinicopathological features of OSCC patients in China.

In the present study, an integrated method to screen *β*-catenin-associated genes (BCAGs) was developed. A flowchart of the analysis pipeline is shown in Figure S1. First, a *β*-catenin-protein interaction network was constructed, and several hub genes were screened out based on differential expression of genes (DEGs), nodes were connected within the network, and the outcome of overall survival (OS) was measured. RUVBL1, also known as Pontin protein, was associated with low survival rates. Therefore, it was postulated that RUVBL1 may serve as a potential diagnostic and prognostic biomarker, as well as a therapeutic target against OSCC, and may help to define the molecular mechanisms of *β*-catenin-induced tumorigenesis in OSCC.

## 2. Materials and Methods

### 2.1. Data Collection and OSCC Patients

OSCC microarray datasets of GSE30784 (OSCC *n* = 164 and adjacent nontumor tissue *n* = 45), GSE37991 (OSCC *n* = 40 and adjacent nontumor epithelial tissue *n* = 40), and GSE31056 (OSCC *n* = 21 and adjacent nontumor tissue *n* = 24) were obtained and downloaded from the NCBI Gene Expression Omnibus (GEO) (https://www.ncbi.nlm.nih.gov/geo/) using the R package, “GEOquery.” R packages “limma” and “ggstatsplot” were used to annotate all probes and produce a differential expression matrix. After data preprocessing, *p* < 0.05 and ∣log_2_ FC | ≥1 (FC, fold change) were defined as the threshold to screen differential expression genes (DEGs). Principal component analysis (PCA) was also performed using “prcomp” in R studio.

### 2.2. Selection of Hub Genes by *β*-Catenin-Protein Interaction Network and Functional Annotation

To identify BCAGs in OSCC, *β*-catenin-associated protein (BCAP) interaction network was constructed through STRING and IMEx database by Networkanalyst (https://www.networkanalyst.ca/). Venn plot (http://bioinfogp.cnb.csic.es/tools/venny/index.html) was visualized by identifying genes that intersected among DEGs and genes from STRING and IMEx. The selected DEGs were further used to construct a *β*-catenin-associated protein interaction network using Networkanalyst and visualized by Cytoscape [13]. After the rank of node degree, node genes (node degree > 10) were considered as hub genes associated with *β*-catenin. Hub gene expression was also shown by box plot using GSE datasets. Correlation analysis between *β*-catenin and hub gene expression was analyzed by Pearson's correlation analysis using HNSC data from the Gene Expression Profiling Interactive Analysis (GEPIA) (http://gepia.cancer-pku.cn/) database. A *p* value < 0.05 was considered significant. BCAGs within the PPI network were annotated and analyzed using the Kyoto Encyclopedia of Genes and Genomes (KEGG), Reactome pathway database, and Gene Ontology (GO) with default parameters through Metascape (https://metascape.org/gp/index.html#/main/step1). *p* values< 0.05 and FDR < 1 were considered significant.

### 2.3. Overall Survival Analysis

The effects of hub genes on OS were analyzed using the Mantel-Cox test and shown in a heat map created by GEPIA. The Kaplan-Meier curves were used to analyze the survival outcome of HNSCC patients with relatively high or low expression of selected hub genes on GEPIA. Log-rank *p* < 0.05 was considered significant.

### 2.4. Immunohistochemistry (IHC)

Tissue microarray of OSCC (Cat # TC0224) including 50 OSCC samples and ten normal oral tissue samples was purchased from the Auragene Bioscience Company (Changsha, China). RUVBL1 protein levels were detected by tissue microarray using anti-RUVBL1 antibody (Cat # sc-393905, Santa Cruz, Shanghai, China) followed by an SV-DAB two-step kit (Cat # SV0004, Boster Biological Technology Company, China). Several serial 4 *μ*m thick sections were cut and mounted on silanized slides. After several steps of deparaffinizing, rehydrating, and blocking with endogenous peroxidase, the sections were rinsed with 0.01 M PBS and incubated in blocking buffer (0.01 M PBS, 0.3% Triton X-100, and 5% normal goat serum) for 30 min. The slides were then exposed to 1 : 100 primary anti-RUVBL1 antibody at 4°C overnight. After further incubation with the secondary HRP-antibody (Cat # BA1055, Boster, China) for 30 min at 37°C, the slides were washed five times with PBS. The chromogenic reaction was performed using freshly prepared diaminobenzidine (DAB) for 10 min and Harris hematoxylin. Hamamatsu NanoZoomer RS Digital Pathology System image analysis software was used to analyze the pathological images. Two clinical pathologists independently examined and evaluated all IHC staining results.

### 2.5. Colony Formation Assay

The OSCC cell line, H157 (Cat # 3153C0001000000260), was obtained from Kunming Cell Bank of Chinese Academy of Sciences and cultured in DMEM/F12 medium with 10% fetal bovine serum (FBS). Small interfering RNA (siRNA) was purchased from GenePharma (Shanghai, China), and the sequences are listed in Table S1. After seeding in a 6-well culture plate for 24 h, the H157 cells were transfected with 100 nM siRNA of RUVBL1 and scramble RNA (siNC) by Lipofectamine 2000. Lipofectamine 2000 was also used as a vehicle control. The medium was replaced every three days, and transfection was performed again after nine days. The H157 cells were stained with 0.05% crystal violet and washed twice with 1×PBS.

### 2.6. Wound-Healing Assay

The Cal-27 cell line was purchased from the Cell Resource Center, Chinese Academy of Medical Sciences, and cultured in DMEM-H medium with 10% FBS. The H157 and Cal-27 cell lines were seeded in culture insert (ibidi) with DMEM medium containing 5% FBS and grown to 90% confluence to evaluate the migration ability. The culture inserts were removed before the straight scratch in each culture insert was made. After washing twice with 1×PBS, the cells were transfected with siRUVBL1 or siNC for 24 h, respectively, as described above. The cells that migrated to the scratched area were examined under a microscope after 48 h.

### 2.7. Cell Invasion Assay

A cell invasion assay was used to assess the invasive ability of the H157 and Cal-27 cells in a transwell chamber (8 *μ*m pores, Costar, USA, Cat #. 3422). After transfecting the cells with siRNA, the H157 cells were seeded in the upper chamber with Matrigel matrix (BD Biosciences) and serum-free DMEM/F12 medium. The lower matched 6-well plates contained DMEM/F12 medium supplemented with 10% FBS. After a 48 h incubation, the H157 and Cal-27 cells that had invaded the lower membrane surface of the upper chamber were stained with crystal violet (0.05%), scanned, and examined by microscope.

### 2.8. Western Blotting

After two independent treatments with siRUVBL1 or siNC, the H157 and Cal-27 cells were incubated and lysed with RIPA buffer. The total protein was isolated, and the concentrations were determined using the bicinchoninic acid (BCA) method (Cat# B0009, Beyotime, China). Standard western blotting was performed, and after sodium dodecyl sulfate-polyacrylamide gel electrophoresis (SDS-PAGE), the protein was transferred from the gel onto polyvinylidene fluoride (PVDF) membranes. PVDF membranes were blocked using blocking solution (Cat# AR0004, Boster, China) and incubated with anti-*β*-catenin, anti-RUVBL1, anti-vimentin (Cat# D21H3, Cell Signaling Technology, Shanghai, China), anti-E-cadherin (Cat# 24E10, Cell Signaling Technology), or anti-*β*-actin antibodies (Cat# A1978, Sigma-Aldrich, Shanghai, China). Immunoreactive proteins were detected by chemiluminescence, and the relative intensity of the gray band was calculated using ImageJ.

### 2.9. Statistical Analysis

RUVBL1 expression in each group of clinical traits was compared by chi-square test using SPSS version 17.0 (SPSS, Inc., USA). *Χ*^2^ was the chi-square value. ∗A value of *p* < 0.05 was considered significant.

## 3. Results

### 3.1. Construction of *β*-Catenin-Protein Interaction Network and Functional Annotation

**Figure 1:**
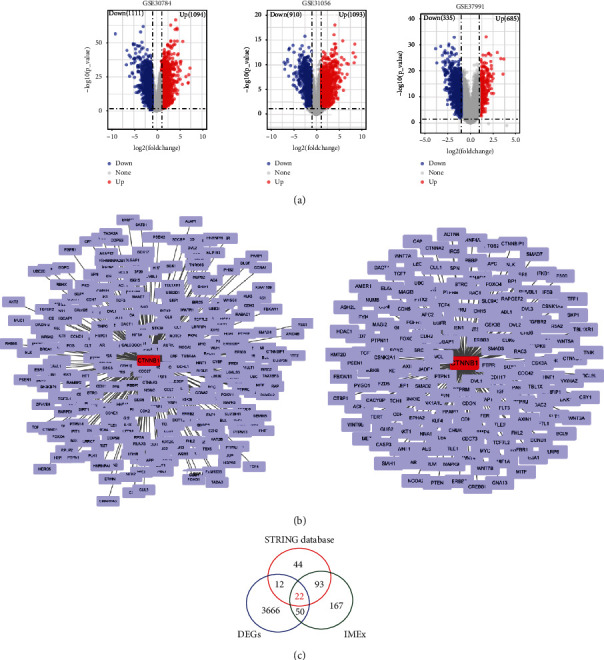
Screening of *β*-catenin-associated genes based on *β*-catenin-protein interaction and differential gene expression in OSCC microarrays. (a) Volcano plot visualizing gene expression in three OSCC microarray datasets. (b) *β*-catenin-protein interaction shown in the STRING and IMEx databases. (c) Venn diagram identifying DEG and *β*-catenin-associated genes that overlap in the STRING and IMEx databases.

**Figure 2:**
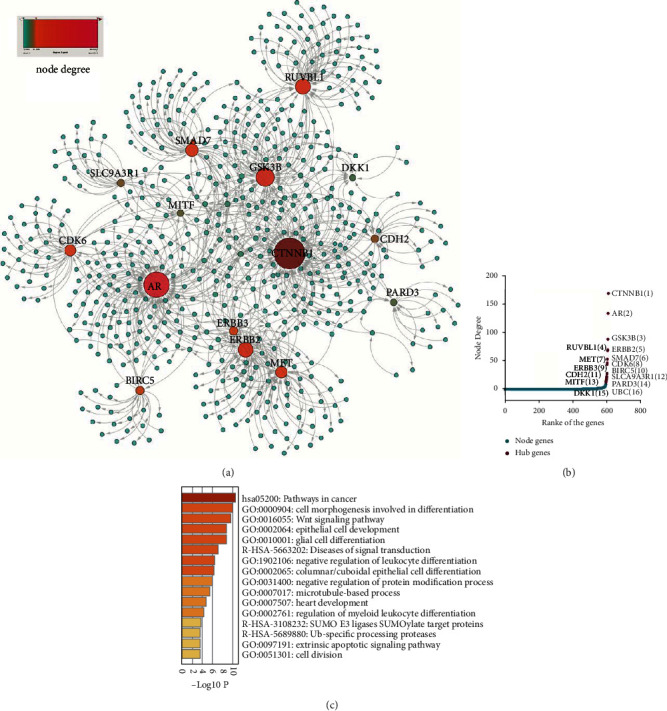
Screening *β*-catenin-associated hub genes through PPI interaction and rank of the node degree. (a) *β*-catenin and protein interaction network. (b) The rank of node degree for node genes involved in the *β*-catenin-protein network. (c) Functional annotation of genes involved in the network by KEGG, GO, and Reactome pathways on Metascape.

Three public OSCC microarray datasets were used to obtain DEGs in OSCC patients. Principal component analysis (PCA) showed that normal and OSCC samples could be clearly separated (Figure 1(a)). Differential expression genes (DEGs) in the three datasets are shown in a volcano plot (Figure 1(b)). Three groups of DEGs from three datasets were combined into one DEG profile. To screen hub genes involved in *β*-catenin signaling, a BCAP interaction network was constructed, and 170 and 331 genes from STRING and IMEx, respectively, were screened out (Figure 1(c)). Twenty-two genes were selected based on the intersection of DEGs and BCAGs from STRING and IMEx (Figure 1(d)). These genes were used to construct a *β*-catenin-protein interaction network (Figure 2(a)). Based on the rank of node degree for genes involved in the network, the top fourteen hub genes (node degree > 10) were selected (Figure 2(b)).

To characterize the molecular function of node genes involved in the BCAP network, hub genes were annotated on Metascape (Figure 2(c)). The results of KEGG, Reactome, and GO pathway analyses showed that many hub genes involved in *β*-catenin signaling were enriched in cancer-related pathways. Some genes were involved in vital signaling pathways, such as cell morphogenesis required for differentiation that occurs via Wnt signaling. Other hub genes are critical for cancer cell survival and growth, playing an important role in epithelial and glial cell development, cell division, and microtubule-based processes.

### 3.2. Screening of Hub Genes Involved in the *β*-Catenin-Protein Network

**Figure 3:**
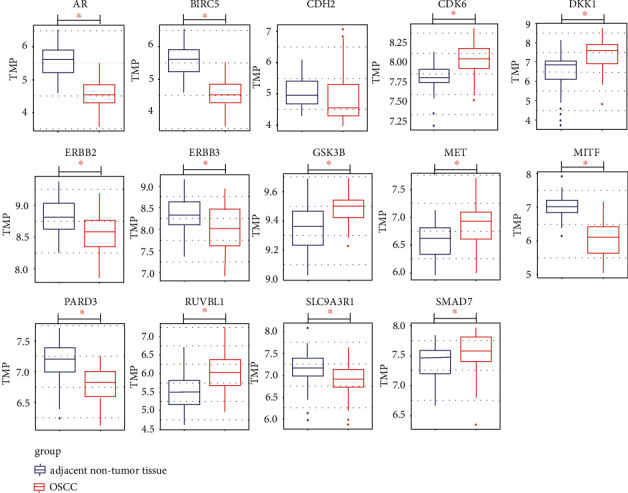
Box plot of *β*-catenin-associated hub gene expression in GSE37991. ^∗^*p* value < 0.05 indicates a significant difference between the OSCC and normal tissue groups.

**Figure 4:**
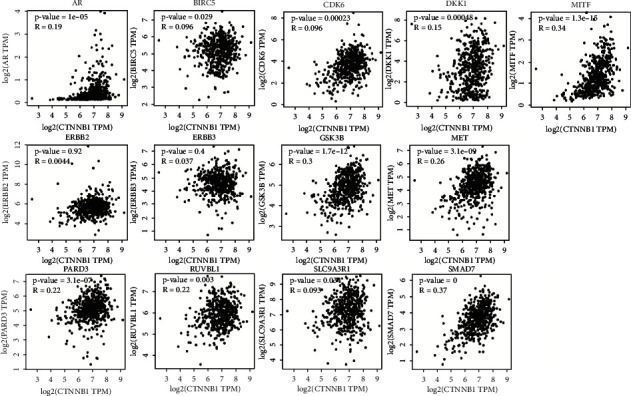
Pearson's correlation analysis was used to assess the association between *β*-catenin and hub gene expression using HNSC data from the GEPIA database. A *p* value < 0.05 was considered significant. *R* represents Pearson's correlation coefficient.

Using GSE37991, six overexpressed and seven downregulated hub genes were found in OSCC versus adjacent nontumor epithelial tissue (Figure 3). Two other microarrays were used to analyze differential hub gene expression (Figures S2 and S3). The Pearson correlation analysis on GEPIA showed that the expression of 12 genes correlated positively with *β*-catenin expression (*p* < 0.05) (Figure 4). *BIRC5* and *SLC9A3R1* correlated weakly (*R* < 0.2), *AR*, *CDH2*, *CDK6*, *DKK1*, *MET*, *PARD3*, and *RUVBL1* correlated moderately (*R* < 0.3), *GSK3B*, *MITF*, and *SMAD7A* associated strongly (*p* < 0.05, *R* > 0.3), and *ERBB2* and *ERBB3* were not associated with *β*-catenin expression (*p* > 0.05, *R* < 0.1). These findings suggest that aberrant expression of *β*-catenin correlates with expression of the 12 hub genes. *CDH2* expression did not change between the normal and OSCC tissue. Eleven genes were used in the following survival analysis except for *ERBB2*, *ERBB3*, and *CDH2*.

### 3.3. Association between Overall Survival (OS) and Hub Gene Expression

**Figure 5:**
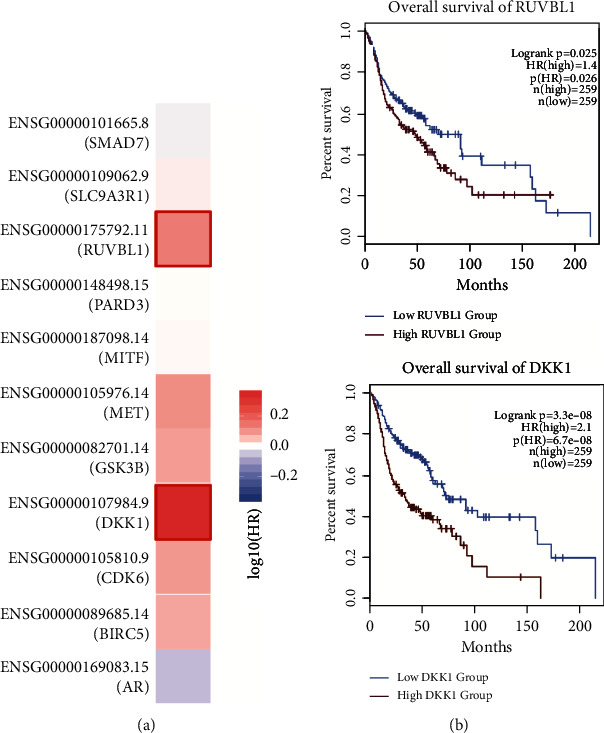
OS analysis of OSCC patients with high or low hub gene expression. (a) Survival heat map for 14 selected hub genes. (b) Kaplan-Meier curve analysis for hub gene correlation with overall survival in OSCC patients. A log-rank *p* value < 0.05 was considered significant.

Eleven hub genes, including *SMD7*, *SLC9A3R1*, *RUVBL1*, *PARD3*, *MITF*, *MET*, *GSK3B*, *DKK1*, *CDK6*, *BIRC5*, and *AR*, were used to assess the effect on overall survival using the Mantel-Cox test in GEPIA. An OS heat map showed that log_10_ HR values of *DKK1* and *RUVBL1* were higher than 0.2 (Figure 5(a)). The Kaplan-Meier curve also showed that high levels of *DKK1* and *RUVBL1* correlated significantly with poor survival outcomes (log-rank *p* < 0.05) (Figure 5(b)). These results imply that *DKK1* and *RUVBL1* can predict OS.

### 3.4. RUVBL1 Is a Hub Gene Involved in *β*-Catenin Signaling in OSCC

**Figure 6:**
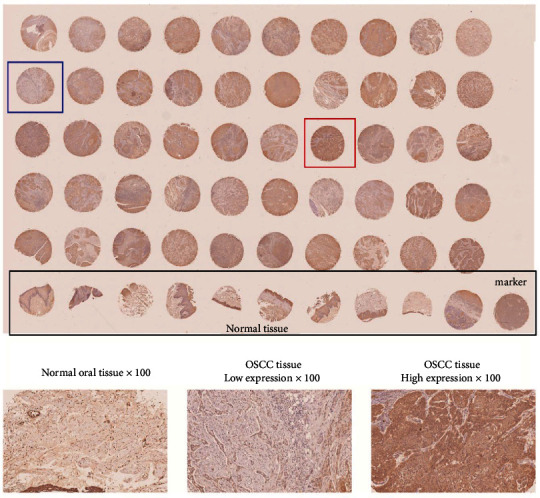
Tissue microarray analysis using IHC to detect RUVBL1 expression in OSCC tissue. The black box indicates normal oral tissue, the blue box represents low expression of RUVBL1, and the red box represents high expression of RUVBL1.

*RUVBL1* functions as a component of several chromatin-remodeling complexes and plays an essential role in transcriptional regulation, DNA damage repair, and telomerase activity [14]. In the initial screen of hub genes related to *β*-catenin, overexpression of *RUVBL1* was found in the GSE37991 dataset of OSCC. To further identify the results, a tissue microarray of OSCC samples was used to analyze *RUVBL1* expression. Compared to normal oral tissue, RUVBL1 expression was primarily higher in the cytoplasm and nucleus of OSCC (Figure 6). To assess the relationship between RUVBL1 expression and clinical characteristics, RUVBL1 expression was divided into high (*n* = 25) and low (*n* = 25) groups. A relationship analysis was conducted by chi-square, and RUVBL1 expression was correlated with histological grade (Table 1).

**Figure 7:**
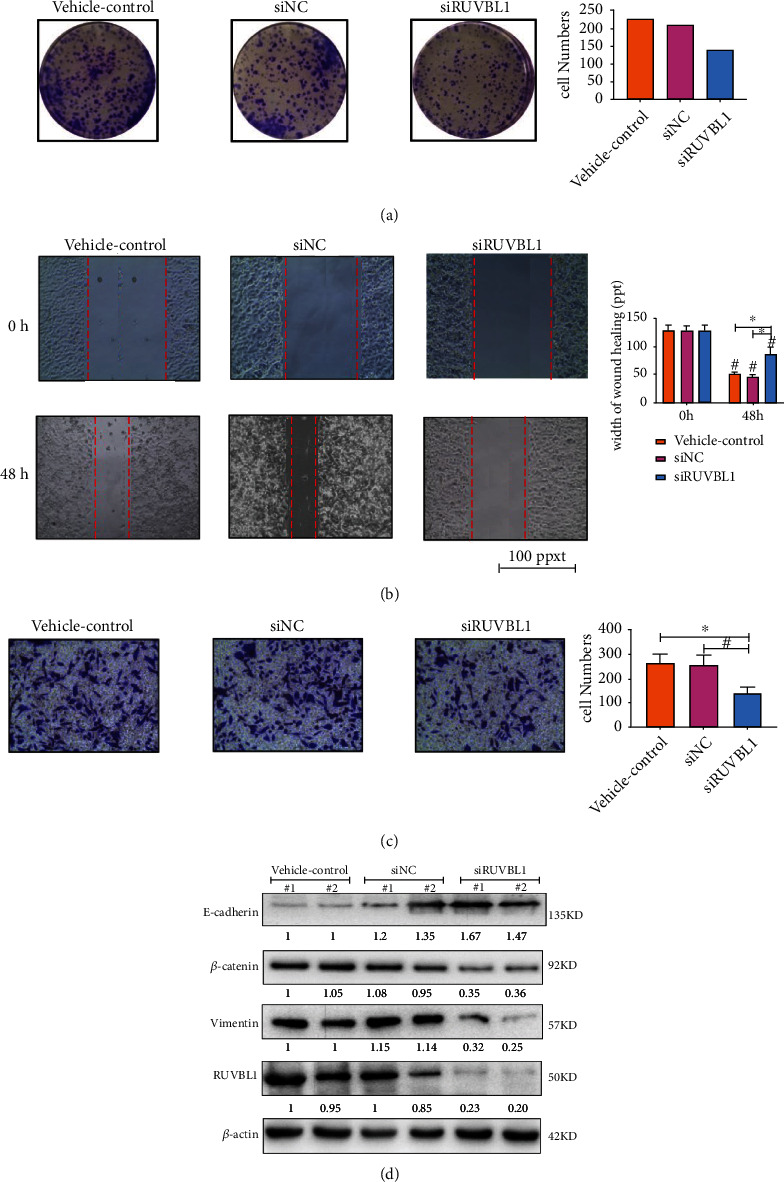
Knock-down of RUVBL1 by siRNA attenuated growth and metastasis of the OSCC cell line, H157, by inhibiting *β*-catenin signaling. (a) Knock-down of RUVBL1 by siRNA suppressed H157 cell proliferation as indicated using the colony formation assay. The box plot showed the number of cells. (b) Knock-down of RUVBL1 by siRNA inhibited migration of the H157 cells in the wound healing assay. Significant differences between groups were assessed using a Student's *T* test and shown by ^∗^ and ^#^, *p* < 0.05. (c). After transfection of the H157 cells with RUVBL1 siRNA or scramble siRNA (siNC), cell invasive ability was determined by cell invasion assay. All experiments were performed in triplicate. Significant differences between groups were assessed using a Student's *T* test and shown by ^∗^ and ^#^, *p* < 0.05. (d) Expression of *β*-catenin pathway proteins was detected by western blot analysis. Intensity of the gray band was calculated by ImageJ and shown in the figure.

**Figure 8:**
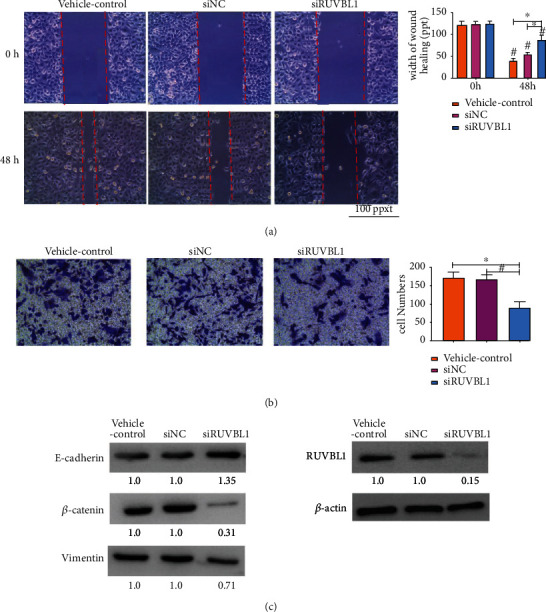
Knock-down of RUVBL1 by siRNA attenuated growth and metastasis of the OSCC cell line, Cal-27, by inhibiting *β*-catenin signaling. (a) Knock-down of RUVBL1 by siRNA inhibited migration of the Cal-27 cells in the wound healing assay. Significant differences between groups were assessed using a Student's T test and shown by ∗ and #, *p* <0.05. (b). After transfection of the Cal-27 cells with RUVBL1 siRNA or scramble siRNA (siNC), cell-invasive ability was determined by cell invasion assay. All experiments were performed in triplicate. Significant differences between groups were assessed using a Student's *T* test and shown by ^∗^ and ^#^, *p* < 0.05. (c) Expression of *β*-catenin pathway proteins was detected by western blot analysis. Intensity of the gray band was calculated by ImageJ and shown in the figure.

Next, the ability of OSCC cell lines to grow and metastasize was measured after the knock-down of RUVBL1 by siRNA transfection. The colony formation assay showed that RUVBL1 siRNA resulted in less H157 cell growth and smaller colony size than the scrambled siRNA (siNC) and vehicle control (Figure 7(a)). The results from the wound healing assay indicated that the gaps of wound healing were getting close after 48 h (^∗^*p* < 0.05), but the suppression of RUVBL1 by siRNA delayed the growth and migration of the H157 cells and Cal-27 cells (^#^*p* < 0.05) (Figures 7(b) and 8(a)). In the cell invasion model of transwell, knock-down of RUVBL1 by siRNA in the H157 and Cal-27 cells significantly prevented cell invasion compared to transfection with siNC and the vehicle control (*p* < 0.05) (Figures 7(c) and 8(b)). Since RUVBL1 was shown to play a role in the *β*-catenin pathway, related protein expression was measured after siRNA transfection. RUVBL1, *β*-catenin, and vimentin all decreased in both H157 and Cal-27 cells after transfection with siRUVBL1, compared to transfection with siNC and vehicle control (Figures 7(d) and 8(c)). Moreover, E-cadherin expression was elevated in the H157 cells exposed to siRUVBL1. These results imply that knock-down of RUVBL1 by siRNA inhibited OSCC cell growth and metastasis, likely by suppressing *β*-catenin signaling.

## 4. Discussion

While surgical and chemotherapy strategies to treat OSCC have improved significantly, the diagnosis and prognosis of OSCC patients require further investigation. Numerous studies have indicated that cancer cell mutations can promote cell proliferation and metastasis in several cancer types, including OSCC [15, 16]. Wnt/*β*-catenin signaling was shown to play a vital role in the tumorigenesis of malignant tumors which promoted an investigation into whether *β*-catenin was expressed in the nucleus of OSCC cells [6, 17]. Indeed, beta-catenin expression was found in 87% of well-differentiated OSCC squamous cells and was significantly associated with histological grade (*p* < 0.001) [6]. Other studies showed that nuclear *β*-catenin was expressed in 35.9% of adjacent and 44.4% of “distant” OSCC samples [12]. Dysregulation of miRNA 34a-5p [18] and miR-106a [19], lncRNA MINCR [20], FTH1P3 [21], CCAT1 [22], and MEG3 [23] activated Wnt/*β*-catenin signaling to promote cell proliferation and migration in OSCC. Evidence indicates that targeting *β*-catenin using specific inhibitors and siRNA abrogated *β*-catenin-induced malignant growth of OSCC and enhanced sensitivity to chemotherapeutics [24–28].

Given the aberrant activation of *β*-catenin signaling in OSCC, screening for *β*-catenin-related proteins that can serve as novel biomarkers is essential to better understand this disease. Based on the differential expression of genes in OSCC and *β*-catenin-associated genes in the *β*-catenin-protein network, 22 genes were selected from the intersection of DEGs and *β*-catenin-associated genes in the STRING and IMEx databases. These 22 potential *β*-catenin-associated genes were used to construct a *β*-catenin-protein network, and the top 14 genes (node degree > 10) were chosen. Signaling pathway enrichment analysis indicated that many hub genes were enriched in cancer-related pathways and essential signaling pathways related to cell growth, survival, and differentiation. Pearson's correlation analysis was performed, and the results showed that 12 hub genes containing *AR*, *BIRC5*, *CDH2*, *CDK6*, *DKK1*, *GSK3B*, *MET*, *MITF*, *PARD3*, *RUVBL1*, *SLC9A3R1*, and *SMAD7* were significantly related to *β*-catenin expression, except *ERBB2* and *ERBB3*. Eleven genes were used to analyze overall survival except for *ERBB2*, *ERBB3*, and *CDH2*.

To determine whether hub BCAG expression was a prognostic indicator for OSCC, an overall survival heat map of 11 hub genes analyzed using the Mantel-Cox test showed that only two genes influenced overall survival (log-rank *p* < 0.05). The Kaplan-Meier curve also showed that high expression of *DKK1* and *RUVBL1* was associated with poor survival outcomes. Many studies have shown that *DKK1* plays various roles in Wnt signaling during several cancers [29–32], which correlates with the overall survival of OSCC patients [33]. While *DKK1* is frequently upregulated in OSCC-derived cell lines and primary OSCCs compared with normal cells, *DKK1*-positive patients have a low risk of regional lymph node metastasis [29]. Further study is necessary to interpret these conflicting findings and better understand the relationship between *DKK1* and the clinical traits of OSCC.

The present study focused on RUVBL1, another *β*-catenin-associated protein. RUVBL1, known as TIP49 or Pontin, exhibits weak ATPase activity. This gene is an essential component of the TIP60 complex [34], which accompanies and modulates transcription factors, including MYC, E2F1, and *β*-catenin, and is involved in chromatin remodeling that contributes to carcinogenesis [35]. The upregulation and nuclear localization of RUVBL1 along with *β*-catenin are highly correlated with colorectal carcinoma progression, which enhances TCF/*β*-catenin-mediated transcription of Wnt target genes and may thus promote carcinogenesis [36, 37]. Non-small-cell lung cancer (NSCLC) patients with poor survival outcomes also had higher RUVBL1 and RUVBL2 expression in NSCLC [38]. Notably, a RUVBL1 mutant protein, TIP49-D302N, interacted with *β*-catenin and occupied the position of RUVBL1, thus inhibiting *β*-catenin-mediated activation of TCF-dependent neoplastic cellular genes [39]. Inhibition of the *β*-catenin/TCF-dependent genes, ITF2 and Axil, was seen using small interfering RNA against endogenous RUVBL1 [39].

The findings indicated that RUVBL1 was overexpressed in the cytoplasm and nucleus of OSCC cells by IHC. In addition, RUVBL1 expression was significantly related to the histological grade of OSCC (*p* = 0.005). Pearson's relation analysis showed that RUVBL1 was positively related to *β*-catenin (*p* = 0.003) in HNSC data of TCGA using GEPIA (Figure 4). Thus, RUVBL1 is likely to be a positive modulator of *β*-catenin signaling. Knock-down of RUVBL1 by siRNA was shown to contribute to the lower cell number and small size of the H157 cell colony compared to cells transfected with scrambled siRNA (siNC) and vehicle control. Fewer cells were transfected with siRUVBL1 on the membrane of the upper chamber than were transfected with siNC and vehicle control, indicating that knock-down of RUVBL1 significantly decreased the invasive ability of the H157 cells. Involvement of RUVBL1 in the *β*-catenin signaling pathway showed that RUVBL1, *β*-catenin, and vimentin expression were reduced in the H157 cells. It was also found that RUVBL1 suppression by siRNA could decrease the migration and growth of the Cal-27 cells. Interestingly, E-cadherin expression was elevated in the H157 cells exposed to siRUVBL1. These results suggest that RUVBL1 knock-down inhibited the growth and metastasis of an OSCC cell line by inactivating *β*-catenin signaling. Recently, RUVBL1, a component of R2TP, a well-conserved molecular chaperone complex, was shown to be required for HSC-4 cell proliferation and migration [40]. The present study provides additional evidence that RUVBL1 enhances the invasive and migration ability of OSCC cell lines, likely by interacting and activating *β*-catenin-driven transcription. More in-depth experiments of the molecular mechanisms of RUVBL1 in OSCC require future investigation.

## 5. Conclusions

RUVBL1 expression is a favorable biomarker for predicting the overall survival of OSCC patients. In addition, knock-down of RUVBL1 in the H157 cells showed that this gene is a potential diagnostic and prognostic biomarker, as well as a target for OSCC treatment. Further investigation of RUVBL1 may reveal additional molecular mechanisms of *β*-catenin-driven OSCC tumorigenesis.

## Figures and Tables

**Table 1:** Association between RUVBL1 expression and clinical characteristics.

Variable		RUVBL1			
	Total	Low	High	*p* value	*Χ* ^2^
Age				0.189	1.389
≤50	18	11	7		
>50	32	14	18		
Gender				0.325	0.388
Male	28	13	15		
Female	22	12	10		
Histological grade				0.005^∗^	8.084
G1	38	23	15		
G2+G3	10	1	9		
Stage				0.095	3.030
I+II	44	24	20		
III+IV	6	1	5		
TNM stage				0.387	0.333
T1	20	11	9		
T2+T3	30	14	16		

*Χ* ^2^ was chi-square value. ^∗^A value of *p* < 0.05 was considered as significant difference.

## Data Availability

The data used to support the findings of this study are available from the NCBI Gene Expression Omnibus (GEO) (https://www.ncbi.nlm.nih.gov/geo/), and the code would be supplied from the corresponding author upon request.

## Supplementary Materials

Supplementary Materials Figure S1: an overall flowchart about the analysis pipeline. Figure S2: expression of genes in GSE30784. Box plot of *β*-catenin-associated hub gene expression in GSE30784. ^∗^*p* value < 0.05 indicates a significant difference between the OSCC and normal tissue groups. Figure S3: expression of genes in GSE31056. Box plot of *β*-catenin-associated hub gene expression in GSE31056. ^∗^*p* value < 0.05 indicates a significant difference between the OSCC and normal tissue groups. Table S1: the sequences of siRNA of RUVBL1 and scramble control.
