# Supplementary material for: Involvement of RUVBL1 in WNT/β-Catenin Signaling in Oral Squamous Cell Carcinoma
Source: Dis Markers. 2022 Apr 22;2022:3398492. doi: 10.1155/2022/3398492 (PMC9054432; doi:10.1155/2022/3398492)
Supplement: Supplementary Materials — Figure S1: an overall flowchart about the analysis pipeline. Figure S2: expression of genes in GSE30784. Box plot of β-catenin-associated hub gene expression in GSE30784. ∗p value < 0.05 indicates a significant difference between the OSCC and normal tissue groups. Figure S3: expression of genes in GSE31056. Box plot of β-catenin-associated hub gene expression in GSE31056. ∗p value < 0.05 indicates a significant difference between the OSCC and normal tissue groups. Table S1: the sequences of siRNA of RUVBL1 and scramble control. [file 3398492.f1.docx]

**Supplementary Materials**

Figure S1. An overall flowchart about the analysis pipeline.


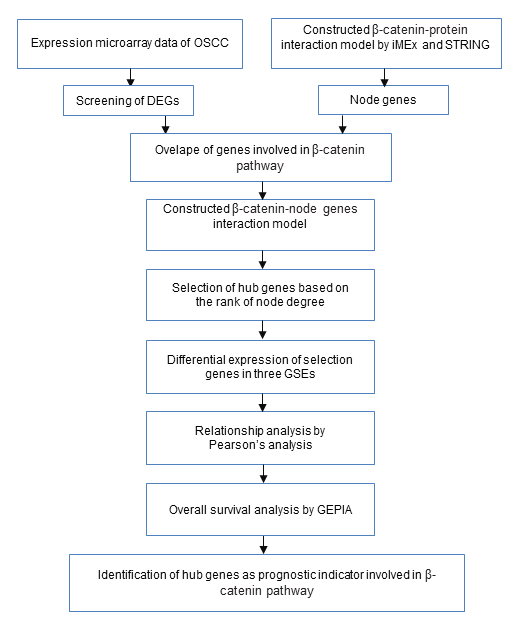


Figure S2. Expression of genes in GSE30784. Box plot of β-catenin-associated hub gene expression in GSE30784. * *P*-value <0.05 indicates a significant difference between the OSCC and normal tissue groups.


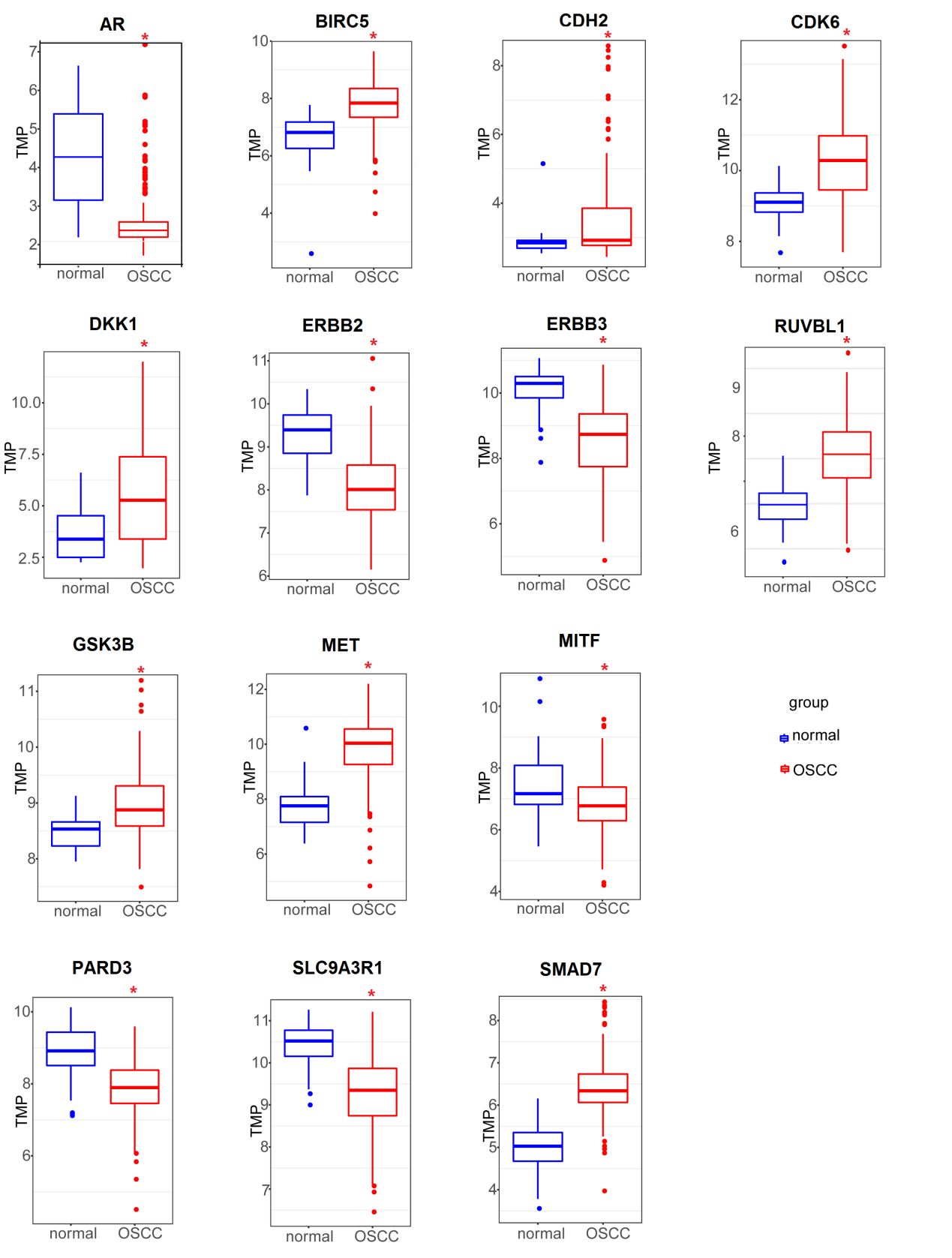


Figure S3. Expression of genes in GSE31056. Box plot of β-catenin-associated hub gene expression in GSE31056. * *P*-value <0.05 indicates a significant difference between the OSCC and normal tissue groups.


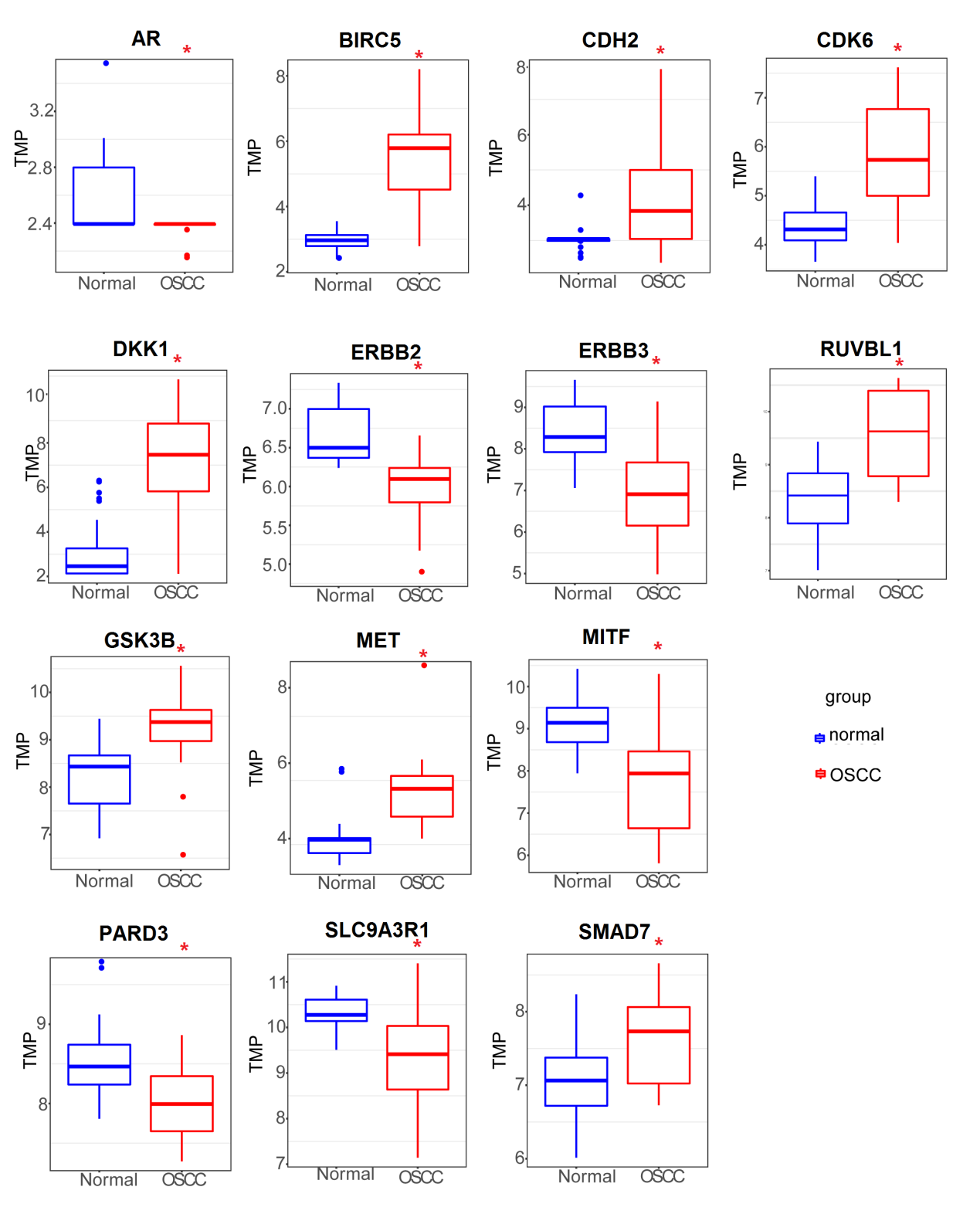


Table S 1. The sequences of siRNA of RUVBL1 and scramble control.

| Items | Sense (5′-3′) | Antisense (5′-3′) |
| --- | --- | --- |
| RUVBL1 siRNA-884 | GCCAGCUAAUGAAGCCAAATT | UUUGGCUUCAUUAGCUGGCTT |
| RUVBL1 siRNA-458 | CCAUUGGGCUGCGAAUAAATT | UUUAUUCGCAGCCCAAUGGTT |
| RUVBL1 siRNA-731 | CCACAGAAUUCGACCUUGATT | UCAAGGUCGAAUUCUGUGGTT |
| Scramble control siRNA （siNC） | GUAUGACAACAGCCUCAAGTT | CUUGAGGCUGUUGUCAUACTT |
